# Photosynthetic and yield responses of rotating planting strips and reducing nitrogen fertilizer application in maize–peanut intercropping in dry farming areas

**DOI:** 10.3389/fpls.2022.1014631

**Published:** 2022-11-16

**Authors:** Fei Han, Shuqing Guo, Song Wei, Ru Guo, Tie Cai, Peng Zhang, Zhikuan Jia, Sadam Hussain, Talha Javed, XiaoLi Chen, Xiaolong Ren, Mohammad Khalid Al-Sadoon, Piotr Stępień

**Affiliations:** ^1^ College of Agronomy, Northwest A&F University, Yangling, Shaanxi, China; ^2^ Key Laboratory of Crop Physic–ecology and Tillage Science in Northwestern Loess Plateau, Ministry of Agriculture, Northwest A&F University, Yangling, Shaanxi, China; ^3^ State Key Lab of Soil Erosion and Dryland Farming on the Loess Plateau, Institute of Soil and Water Conservation, Northwest A&F University, Yangling, Shaanxi, China; ^4^ Department of Agronomy, University of Agriculture, Faisalabad, Pakistan; ^5^ Department of Zoology, College of Science, King Saud University, Riyadh, Saudi Arabia; ^6^ Wroclaw University of Environmental and Life Sciences, Institute of Soil Science, Plant Nutrition and Environmental Protection, Wroclaw, Poland

**Keywords:** dry farming areas, maize-peanut intercropping, rotation of crop planting strip, N reducing, light adaptation

## Abstract

Improving cropping systems together with suitable agronomic management practices can maintain dry farming productivity and reduce water competition with low N inputs. The objective of the study was to determine the photosynthetic and yield responses of maize and peanut under six treatments: sole maize, sole peanut, maize–peanut intercropping, maize–peanut rotation–intercropping, 20% and 40% N reductions for maize in the maize–peanut rotation–intercropping. Maize–peanut intercropping had no land-use advantage. Intercropped peanut is limited in carboxylation rates and electron transport rate (ETR), leading to a decrease in hundred-grain weight (HGW) and an increase in blighted pods number per plant (N_BP_). Intercropped peanut adapts to light stress by decreasing light saturation point (I_sat_) and light compensation point (I_comp_) and increasing the electron transport efficiency. Intercropped maize showed an increase in maximum photosynthetic rate (Pn_max_) and I_comp_ due to a combination of improved intercellular CO_2_ concentration, carboxylation rates, PSII photochemical quantum efficiency, and ETR. Compare to maize–peanut intercropping, maize–peanut rotation–intercropping alleviated the continuous crop barriers of intercropped border row peanut by improving carboxylation rates, electron transport efficiency and decreasing I_sat_, thereby increasing its HGW and N_BP_. More importantly, the land equivalent ratio of maize–peanut rotation–intercropping in the second and third planting years were 1.05 and 1.07, respectively, showing obvious land use advantages. A 20% N reduction for maize in maize–peanut rotation–intercropping does not affect photosynthetic character and yield for intercropped crops. However, a 40% N reduction decreased significantly the carboxylation rates, ETR, I_comp_ and Pn_max_ of intercropped maize, thereby reducing in a 14.83% HGW and 5.75% lower grain number per spike, and making land-use efficiency negative.

## 1 Introduction

China has a big population and little land, relatively sparse vacancy of land resources, and inadequate cultivated land reserve resources (Yang and Li, 2000; Wu et al., 2017). In order to safeguard food security, China has historically advocated intensive cultivation with high input and high output (Yu and Lu, 2006; Jiang et al., 2013). The long-term implementation of the intensive cultivation strategy inevitably brings a negative influence on soil, results in a huge waste of water and nitrogen, and leads to soil desiccation and pollution (Gianfreda et al., 2005), especially in dry farming areas with weak environmental carrying capacity (Yan et al., 2017). Wheat (*Triticum aestivum* L.) and maize (*Zea mays* L.) rotation are the main cropping system in China’s dry farming areas. Studies have shown that the multi-year continuous cropping of maize and wheat declines the quality of cultivated land (Mrabet et al., 2001; Parihar et al., 2017). At present, many farmers in dry farming areas report various degrees of land degradation, including reduced soil fertility, shallow cultivation layers, and poor soil tillage, leading to over-reliance on chemical fertilizers, less risk tolerance, and a decline in farming efficiency.

Land fallow is an important measure to restore fertility, improve grain production capacity, and resist natural disasters in dry farming areas (Gozubuyuk et al., 2014; Nittaya et al., 2017). In addition, land fallow during the dry season in dry crop areas reduces irrigation pressure on farmland in other cropping systems, which can contribute to ensuring regional food security. However, the fall in farm income has hindered the spread of land fallow. Combining cereal-legume intercropping with land fallow will improve farm output due to low agricultural inputs and high economic benefits (Kermah et al., 2017; Rahman et al., 2021). More importantly, intercropping in dry farming can reduce N pollution at the source, as fallow and legumes require far less chemical N fertilizer than cereal. After harvest, legumes leave a large amount of easily decomposable residues and improve the soil microbial environment and soil N mineralization capacity for use by subsequent crops (Stagnari et al., 2017; Kakraliya et al., 2018). It is important to develop a promising and easy-to-operate cropping system with the advantages of crop intercropping and land fallow is of great significance to realize the balanced development of economic and ecological benefits.

Peanut (*Arachis hypogaea* L.) is a widely grown oilseed and cash crop in tropical and subtropical regions, and a major source of oil for human consumption. Maize–peanut intercropping can exert the advantages of marginal effect and peanut biological N fixation, improve the absorption and utilization of nutrient elements in the intercropping population, and reduce replant disease (Li et al., 2009; Guo et al., 2021). Maize makes the largest contribution to the high yield advantage, the adaptation of peanuts to low light is key to determining the economic benefits of intercropping. In a meta-analysis, the first and the third quartile for partial land equivalent ratios (PLER) for peanuts were 0.38 and 0.63 (Feng et al., 2021). Overall, besides the border-row proportion (Wang et al., 2020), the cause of the great differences among PLER was environmental conditions and the N application rate. However, few studies of maize–peanut intercropping focused on dryland farming. In addition, most N reduction studies in intercropping were based on simultaneous reduction of base fertilizer and top dressing, but the key to N reduction in cereal-legume intercropping systems is the efficient use of N by the cereal and the increased nitrogen fixation of the legume in the later growth stages of the crops (Kermah et al., 2017; Yong et al., 2018; Li et al., 2019). Premature N reduction at basal fertilizer is likely to affect maize and peanut growth. It is worth noting that intercropped peanuts are equivalent to long-term continuous cropping in the same area if the planting strips are not changed, and there are barriers to the risk of continuous cropping. To address the above issues, we rotated the maize planting strip (MPS) and peanut planting strip (PPS) and reduce N in maize top dressing based on the previous annual maize–peanut intercropping.

Plants accumulate biomass through photosynthesis, and the photosynthetic utilization capacity of crops is closely related to water, N application rate, and intensity of light interception (Sims et al., 1998b; Coruzzi and Bush, 2001; Flexas et al., 2006). The complex community structure of intercropping systems can alter the distribution and intensity of light and nutrient utilization (Gong et al., 2015; Yang et al., 2017). Lack of water in dry farming areas and N reduction can fundamentally alter photosynthesis in crops, directly affecting dry matter production (Escalona et al., 2012; Chai et al., 2016). We hypothesize that (i) maize-peanut intercropping could increase the farmland yield by modifying the adaptation of peanuts and maize to different light radiation, (ii) rotating the planting strips in intercropping could increase the photosynthetic capacity and yield of peanuts by alleviating continuous cropping obstacles, and (iii) properly decreasing the application of chemical N fertilizer in MPS has no significant effect on photosynthetic capacity and yield of peanuts and maize in maize–peanut intercropping. In testing the hypothesis, we measured (i) soli total N (TN) content in MPS and PPS before planting, 30 days after top dressing (AT_30d_), 60 days after top dressing (AT_60d_), and after harvest, (ii) light interception, light response curve, Rubisco-related enzyme activity, photosynthetic and fluorescence characteristics of peanuts and maize, (iii) yield and yield-related traits under different planting patterns and N application rates. This study provides a theoretical basis for promoting a new intercropping system for peanut and maize in dry farming areas.

## 2 Materials and methods

### 2.1 Site description and experimental design

**Figure 1:**
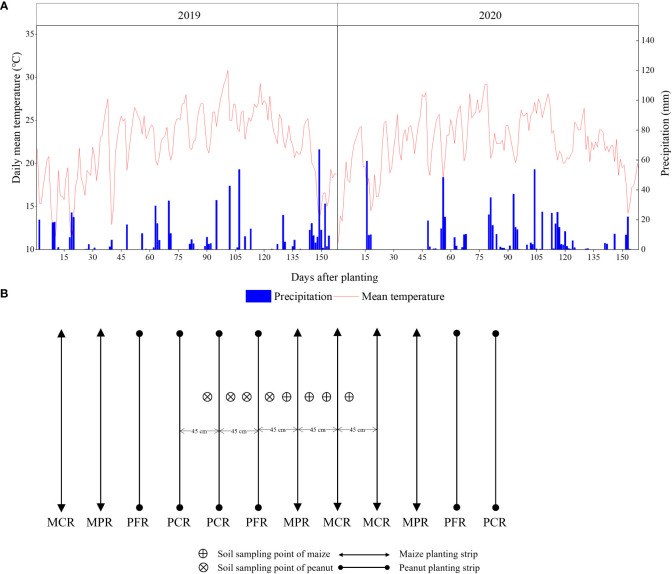
Daily climatic data **(A)** and sample location in intercropping **(B)**.

A three-year field experiment was conducted at the Yangling (108°08′E, 34°17′N), Shaanxi Province, China, from 2018 to 2020. This area has a cold climate with a dry winter and a hot summer. The mean annual temperature was 15°C with mean growing degree days from April to September, an annual sunshine duration of 2440 h, a frost-free period of 200 d, and total annual rainfalls of 993 mm. The climatic data during the study period were presented in **Figure 1A**. The soil was formed from alluvial deposits of the Yellow River and was classified as a sandy loam (Haplic Chernozems classification according to FAO–UNESCO 1988). The soil was sampled for the 0–20 cm depth before sowing in 2018 with 10.01 g kg^−1^ soil organic matter, 0.78 mg kg^−1^ total N, 7.21 mg kg^−1^ available P, and 178.76 mg kg^−1^ available K.

The experiment was established with a randomized, incomplete block design and with three replicates. Seeds of maize (cv. ‘Zhengdan 958’) and peanut (cv. ‘Fenghua No. 5’) were planted by hand on 7 May in rows oriented east to west. In 2018, the experiment comprised five treatments: sole maize, sole peanut, maize–peanut intercropping (I), 20% N and 40% N reductions for maize in the maize–peanut intercropping. In 2019, we rotated the maize planting strip (MPS) and peanut planting strip (PPS) in half of the area in maize–peanut intercropping (I) and all areas in 20% N and 40% N reductions in maize planting strip in the maize–peanut intercropping, and record the numbers RI, RI2, and RI3. In 2020, rotate the MPS and PPS of all RI, RI2, and RI3. Videlicet, 2019 and 2020, there were six treatments: sole maize (M), sole peanut (P), maize–peanut intercropping (I), maize–peanut rotation–intercropping (RI), 20% N (RI2), and 40% N (RI3) reductions in maize planting strip in the maize–peanut rotation–intercropping. The sole maize treatment received N fertilizer at 260 kg N ha^−1^ as urea, of which 90 kg N ha^−1^ of the total N was evenly spread and incorporated into the top 15 cm of soil as base fertilizer, and the remaining N fertilizer was as top dressing at the six-leaf stage of maize. The sole peanut treatment received N fertilizer at 90 kg ha^−1^ N as urea and all were as base fertilizer. Intercropped maize and peanut received the same area–based fertilizer as the corresponding sole crop. For RI2 and RI3, the 20% and 40% N reductions in MPS only took place at the top dressing, that is, 90 kg ha^−1^ was applied to both basal and 118 (RI2) and 66 kg (RI3) ha^−1^ N to the top dressing, respectively.

To soften the soil and eliminate weeds, the soil is mechanically ploughed about 15 cm deep before sowing. The plant spacings were 22.5 and 15 cm for sole maize and sole peanut, and the row spacing for all crops was 45 cm. The stand density was 148,000 plants ha^−2^ for sole peanut and 74,000 plants ha^−2^ for sole maize. The plant spacings and row spacing for intercropped peanut and maize were the same as the sole crop. Therefore, intercropped peanuts and maize had equal row spacing which leads to convenient rotation of planting strips. Two seeds of maize and peanut were planted per hole, and the seedlings were thinned to one vigorous plantlet after the seedling stage. Single plot size was 54 m^2^ (10 m × 5.4 m). Weeds were controlled by manual removal at once when needed. No irrigation water was applied during the growing season.

### 2.2 Measurements

#### 2.2.1 Soil total N content

At preplant, AT_30d_, AT_60d_ and harvest, soil samples were taken with a 9 mm inner–diameter soil drill at 0–20 and 20–40 cm. The intercropping treatment soil samples were divided into peanut and maize planting strip, and each planting strip was sampled between intercropped border and middle row and between intercropped middle rows (**Figure 1B**). Kjeldahl method were employed to measure TN content in the soil (Bremner and Mulvaney, 1996).

#### 2.2.2 Light interception and light response curve

At the R1 stage of maize (full pod formation stage of peanut), the radiation transmission was recorded with a 1 m long line quantum sensor (AccuPAR LP–80, Meter, USA). A set of readings was taken from each crop strip starting at 7:30 and obtained every half hour until 17:30, including the incident and reflected light flux density at the top (canopy), spike leaf, bottom (earth surface) layer of maize, and the canopy, bottom layer of peanuts. Intercropped crops were divided into the intercropped middle row and intercropped border row to record data separately. The integral equation is used to calculate the total received light for the measurement period, while the light interception accumulation (I_I_) is calculated using Eq. 1.


1 $$I_{I}=I_{TI}-I_{TR}-I_{BI}+I_{BR}$$

where I_TI_ means incident I accumulation at the top of crop planting strip, I_TR_ means reflected I accumulation at the top of crop planting strip, I_BI_ means incident I accumulation at the bottom of crop planting strip, I_BR_ means reflected I accumulation at the bottom of crop planting strip.

The light response of photosynthesis for spike leaves of maize and top third leaf of peanut main stems were selected to measure the light response using a portable photosynthetic system (LI–6400, Inc., Logan, NE, USA). In the leaf chamber, the CO_2_ concentration of the sample chamber was stabilized at 400 μmol mol^–1^, and the PPFD was controlled at 0, 50, 100, 200, 300, 400, 600, 800, 1000, 1200, 1400, 1600, 1800, 2000 μmol m^–2^ s^–1^. Selected leaves were acclimated for at least 2 min at each level of PPFD before switching. The Ye model characterizes the Pn–I relationship was Eq. 2 (Ye, 2007; Ye et al., 2013).


2 $$P_{n}=\frac{\alpha \times I\times \left(1-\beta \times I\right)}{1+\text{Υ}\times I}-R_{d}$$

where α means the initial slope of light response curve, R_d_ means the dark respiration rate (μmol (CO_2_) m^–2^ s^–1^), and β and γ are the photoinhibition coefficient and saturation coefficient, respectively (Ye et al., 2013).

The light saturation point (I_sat_, μmol (photon) m^–2^ s^–1^) can be calculated when dPn/dI = 0 as Eq. 3. The light compensation point (I_comp_, μmol (photon) m^–2^ s^–1^) can be calculated when Pn = 0 as Eq. 4. The asymptotic estimate of the maximum photosynthetic rate (Pn_max_, μmol(CO_2_) m^–2^ s^–1^) can be calculated when I = I_sat_ as Eq. 5 (Zhu et al., 2020).


3 $$I_{sat}=\frac{\sqrt{\left(\beta +\gamma \right)/\beta }-1}{\gamma }$$

4 $$I_{comp}=\frac{\alpha -\gamma \times R_{d}-\sqrt{{\left(\alpha -\gamma \times R_{d}\right)}^{2}-4\times \beta \times \alpha \times R_{d}}}{2\times \beta \times \alpha }$$

5 $$Pn_{\text{max}}=\alpha {\left(\frac{\sqrt{\left(\beta +\gamma \right)}-\sqrt{\beta }}{\gamma }\right)}^{2}-R_{d}$$

#### 2.2.3 Photosynthetic and fluorescence characteristics

At the R1 stage (full pod formation stage of peanut), spike leaves of maize and top third leaf of peanut main stems were selected to measure photosynthetic parameters using a Li-6400 portable photosynthesis system (LI–COR Inc., Lincoln, NE, USA). All measurements were conducted from 9:30 to 11:00 h under a CO_2_ concentration of 400 μmol mol^−1^. Intercropped crops were divided into the intercropped middle row and intercropped border row to record data separately. Specific parameters measured included photosynthetic rate (Pn, μmol CO_2_ m^-2^ g^-1^), intercellular CO_2_ concentration (Ci, μmol mol^-1^), and stomatal conductance (Gs, mmol m^-2^ g^-1^).

The leaves for the determination of photosynthesis parameters were taken to determine the Chlorophyll fluorescence parameters with a pulse amplitude modulation 2000 fluorometer (MFMS-2, Hansatech, Kings Lynn, UK) by the method of Zhao and Wang (2002). Specific parameters measured included maximum light energy conversion efficiency of PSII (Fv/Fm), effective quantum efficiency of PSII (ΦPSII), photochemical fluorescence quenching coefficient (qP), non-photochemical fluorescence quenching coefficient (qN), and electron transport rate (ETR).

#### 2.2.4 Rubisco-related enzyme activity

At the R1 stage of maize (full pod formation stage of peanut), 0.1 g fresh leaf samples used for photosynthetic rate determination of 2.2.3 were ground to a powder in liquid nitrogen and then mixed with 2 mL of extraction solution (100 mmol L^-1^ Hepes–Na (pH 8. 0), 10 mmol L^-1^ MgCl_2_, 0.4 mmol L^-1^ EDTA–Na_2_, 1% PVP, 100 mmol L^-1^ Na-ascorbate, 0.1% BSA, and 50 mmol L^-1^ DTT). All extraction fluid was centrifuged for 15 minutes at 12000 × g for 10 min. The supernatant was collected to measure the enzymatic activity.

For Rubisco initial activity, reaction system included 0.1 mL of 5 mmol L^-1^ NADH, 0.1 mL of 50 mmol L^-1^ ATP, 0.1 mL of 0.2 mol L^-1^ NaHCO_3_, 0.7 mL of reactive medium (100 mmol L^-1^ Tris-HCl, 10 mmol L^-1^ MgCl_2_ and 0.4 mmol L^-1^ EDTA–Na_2_), 0.05 mL of 160 U mL^-1^ creatine kinase, 0.05 mL of160 U mL^-1^ phosphoglycerate kinase, 0.05 mL of160 U mL^-1^ Glyceraldehyde–3–phosphate dehydrogenase, 0.15 mL distilled water. The prepared reaction system was shaken well and 630μL was poured into the cuvette. After zeroing with reaction system at 340 nm, 50 μL RuBP and 20 μL enzyme solution were poured into the cuvette and the absorbance was immediately measured every 30 s at 340 nm (Ultraviolet–2600; UNICO Instruments, Shanghai, China) for 3 min. The absolute value of the decrease in absorbance per minute was used to calculate the rubisco initial activity as Eq. 6:


6 $$A_{\text{RuBisCO}}=\frac{\text{Δ}A\times V}{2\times d\times 6.22\times \text{Δ}t\times V_{E}\times m}$$

where A_Rubisco_ (μmol min^-1^ g FW^-1^) means the Rubisco activity; ΔA means the absolute value of the change in absorbance at 340 nm over 3 min; d (cm) means the optical path of cuvette; 6.22 means the absorbance at 340 nm per μmol NADH; 2 means fixation of 1 mol CO_2_ requires 2 mol NADH; Δt means the measurement time; V_E_ means Total volume of the reaction system; m means the fresh sample weight.

For Rubisco total activity, 50 μL of enzyme solution was added to the 100 μL of reactive medium (0.2 mol L^-1^ NaHCO_3_, 100 mmol L^-1^ Tris-HCl, 10 mmol L^-1^ MgCl_2_, and 0.4 mmol L^-1^ EDTA-Na_2_) for 15 min at 30°C. Then, the absorbance was recorded according to the method for determining Rubisco initial activity. Rubisco activation state was expressed as the initial activity divided by the total activity (Butz and Sharkey, 1989).

For Rubisco activase (RCA), 50 μL of enzyme solution and 50 μg of Rubisco passivated with 0.4 mmol L^-1^ RuBP were added to 450 μL of the following reaction solution: 100 mmol L^-1^ Hepes-Na (pH 8. 0), 10 mol L^-1^ NaHCO_3_, 1 mmol L^-1^ EDTA–Na_2_, 10 mmol L^-1^ MgCl_2_, 2.5 mmol L^-1^ DDT, 5 mmol L^-1^ ATP, 5 mmol L^-1^ phosphocreatine, 5 U L^-1^ Creatine Kinase. By adding 25 μL of 12 mmolL^-1^ RuBP after reacting for 10 minutes, the absorbance was determined according to the Rubisco initial activity. RCA activity was the difference between Rubisco activity with and without RuBP passivation.

#### 2.2.5 Yield and yield-related traits

A 43.2 m^2^ (8 m × 5.4 m) plant sample was collected to measure yield per plant, hundred-grain weight (HGW), pods number per plant and blighted pods number per plant (N_BP_) of peanut and yield per plant, hundred-grain weight (HGW) and grain number per spike of maize. LER was used to evaluate the land-use efficiency using a concept proposed by Willey (1979). It is expressed as:


1 $$LER={\text{PLER}}_{\text{M}}+{\text{PLER}}_{\text{P}}=\frac{{\text{Y}}_{\text{IM}}}{{\text{Y}}_{\text{SM}}}+\frac{{\text{Y}}_{\text{IP}}}{{\text{Y}}_{\text{SP}}}$$

where PLER_M_ and PLER_P_ are partial land equivalent ratios of maize and peanut, respectively. Y_SM_ and Y_IM_ are the sole and intercropped maize yields, respectively. Y_SP_ and Y_SP_ are the sole and intercropped peanut yields, respectively.

### 2.3 Data analysis

A factorial set of treatments was arranged within a randomized complete block design. Each treatment was repeated three times for triplicates of sampling. Data were analyzed using Origin 2022. The IBM SPSS Statistic Version 21.0 (StatSoft. Inc.) was used for statistical analysis. Significant differences were tested using an ANOVA in combination with Fisher’s LSD test at P< 0.05 levels. The TN, Rubisco-related enzyme activity, photosynthetic and fluorescence characteristics, yield, and yield-related traits were analyzed independently each year. The values presented in the figures and table were mean + standard errors. In the case of heteroscedasticity, data were analyzed by the Kruskal-Wallis H test and Tamhane’s T2 (P = 0.05) for *post hoc* multiple comparisons.

## 3 Results

### 3.1 Soli total N content

**Figure 2:**
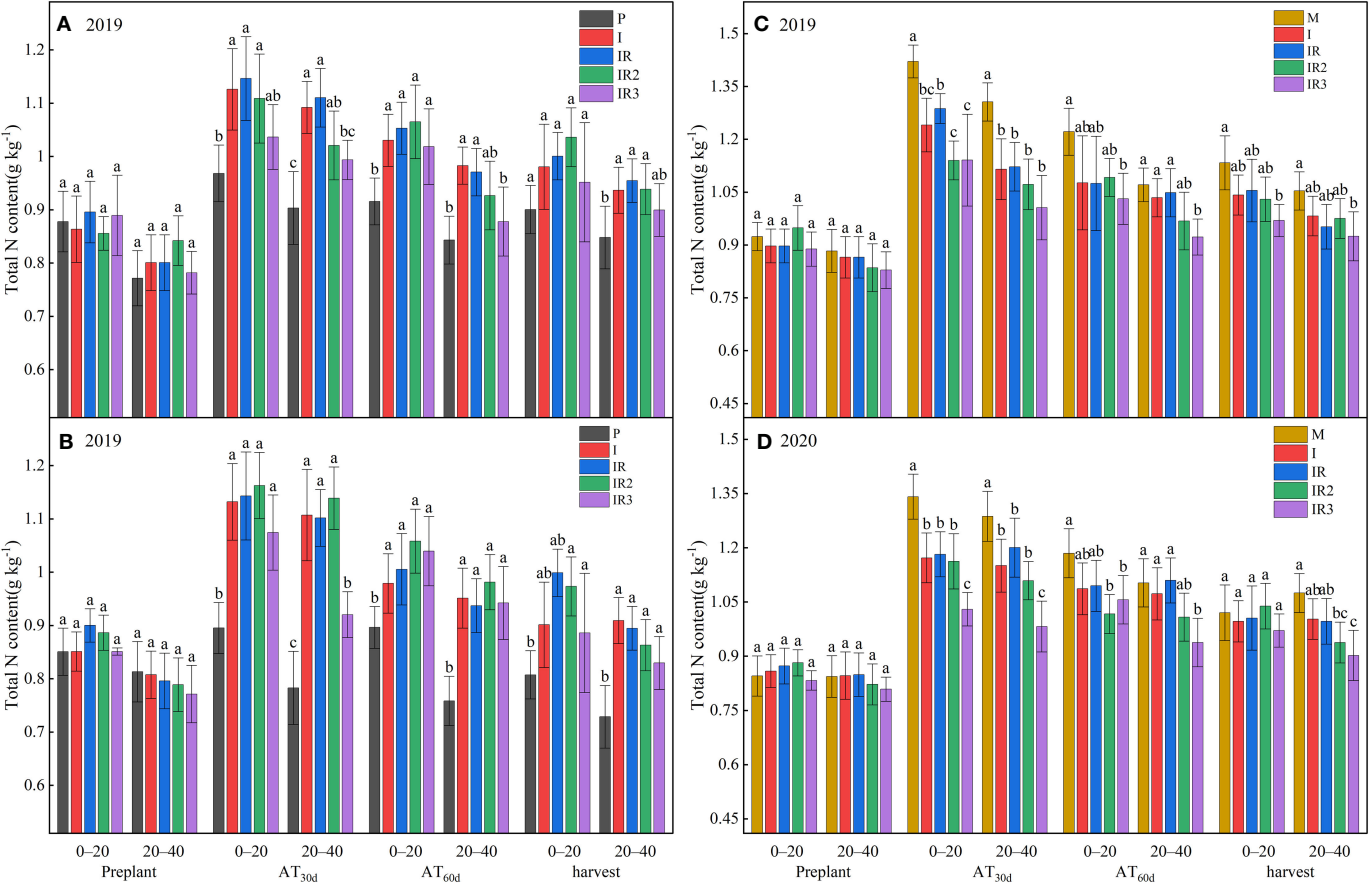
Total N content in 0–20 and 20–40 cm soil in peanut planting strip **(A, B)** and maize planting strip **(C D)**. P, sole peanut; M, sole maize; I, maize–peanut intercropping; RI, maize–peanut rotation–intercropping, RI2, 20% N reduction for maize of RI; ICN3, 40% N reductions for maize of RI. Different lowercase letters represent significant (P<0.05).

Because the top dressing in the intercropping was only in MPS, the soil TN content in MPS in intercropping decreased significantly than sole maize after AT_30d_, and PPS in intercropping increased significantly due to the movement of N in the soil (**Figure 2**). However, at AT_60d_, there was no significant difference between the intercropped MPS and the sole maize, while the soil TN content in MPS was also higher than in the sole peanut.

Rotating the planting strips had no effect on soil TN content (**Figure 2**). A 40% N reduction significantly reduced the soil TN content of MPS in the 0–40 cm at AT_30d_ and the 20–40 cm at AT_60d_. It was also found that the 20–40 cm soil TN content of PPS was reduced by 10.51%–16.48% and 9.56%–9.46% at AT_30d_ and AT_60d_ after 40% N reduction, respectively. A 20% N reduction was only found to reduce the TN content of the MPS by 11.44% at AT_30d_ in 2019.

### 3.2 Photosynthetic character of peanut

**Figure 3:**
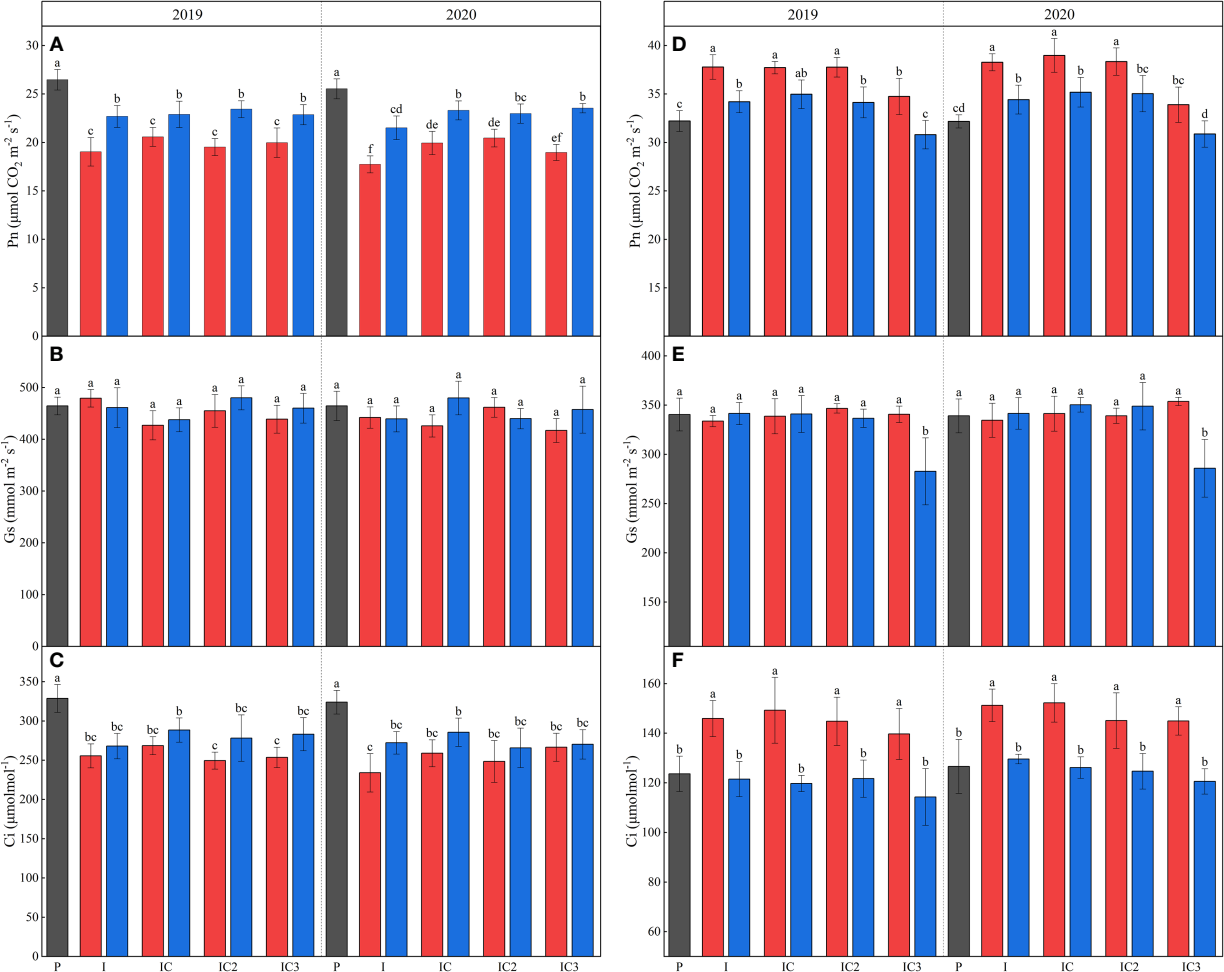
Pn **(A, D)**, Gs **(B, E)** and Ci **(C, F)** for peanut and maize in 2019 and 2020. P, sole peanut; M, sole maize; I, maize–peanut intercropping; RI, maize–peanut rotation–intercropping, RI2, 20% N reduction for maize of RI; ICN3, 40% N reductions for maize of RI. Different lowercase letters represent significant (P<0.05).

During the maize R1 (stem and leaves had finished growing), there was an average reduction of 106.78% and 65.47% in top incident light, 120.40% and 61.28% in bottom incident light and 110.50% and 66.99% in light interception between 2019 and 2020 for intercropped border and middle row peanut compared to sole peanut (**Figures S1**, **S3**; **Table 1**). The intercropped disadvantage caused a significant reduction in Pn and Ci in peanuts (**Figure 3**). The Pn_max_ of the peanut in intercropped middle and border rows in 2019 and intercropped middle row in 2020 were not affected after intercropping, but I_comp_ and I_sat_ showed a decrease, and the decreased degree was greater in intercropped border row of the peanut than in the middle row (**Table 1**). For chlorophyll fluorescence, intercropping increased the ΦPSII and qP, and decreased qN and ETR in peanuts (**Table 2**).

**Table 1:** The Ye model light response curve and light interception of peanut.

Year	Parameters	P	I		RI		RI2		RI3	
BR	MR	BR	MR	BR	MR	BR	MR
	Rd	5.33	4.36	4.92	4.18	5.01	4.28	4.92	4.35	4.75
2019	I_comp_	85.25	65.53	74.57	62.83	75.18	64.72	75.01	65.39	78.81
	I_sat_	1726.13	1631.22	1615.38	1675.83	1618.27	1649.44	1716.50	1655.85	1631.54
	P_nmax_	27.15	27.47	27.54	27.62	27.45	27.62	27.78	27.32	27.58
	R^2^	99.65%	99.79%	99.63%	99.86%	99.60%	99.81%	99.66%	99.80%	99.66%
	I_TI_	21015.47	15234.27	17874.64	15175.49	17748.73	15247.39	17858.22	15353.84	17994.05
	I_DI_	910.85	993.95	950.17	1002.99	947.03	994.17	922.57	979.25	945.40
	I_I_	17702.68	12462.53	14772.52	12461.39	14641.68	12362.78	14714.32	12510.44	14841.65
2020	Rd	5.49	4.82	4.94	4.69	5.06	4.80	5.17	4.80	5.19
	I_comp_	79.44	65.71	70.22	67.19	71.28	67.84	71.24	67.84	70.18
	I_sat_	1781.45	1709.26	1564.57	1576.67	1551.43	1624.45	1561.77	1624.45	1583.95
	P_nmax_	27.47	24.94	28.02	27.57	28.11	27.09	27.43	27.09	27.96
	R^2^	99.47%	99.51%	99.37%	99.13%	99.25%	99.05%	99.52%	98.77%	98.98%
	I_TI_	18225.63	13509.04	14983.51	13415.06	15079.72	13409.28	15158.42	13545.80	15449.77
	I_DI_	952.47	785.67	719.65	794.53	722.52	799.69	729.54	818.19	744.08
	I_I_	16536.09	12294.82	13734.29	12154.05	13767.27	12127.55	13823.79	12210.57	14097.54

P, sole peanut; I, maize–peanut intercropping; RI, maize–peanut rotation–intercropping, RI2, 20% N reduction for maize of RI; ICN3, 40% N reductions for maize of RI.

The parameters are: Dark respiration rate (R_d_, μmol (CO_2_) m^–2^ s^–1^), light saturation point (I_sat_, μmol (photon) m^–2^ s^–1^), light compensation point (I_comp_, μmol (photon) m^–2^ s^–1^), asymptotic estimate of the maximum gross photosynthetic rate (P_gmax_, μmol(CO_2_) m^–2^ s^–1^). The light includes top incident light accumulation (I_TI_, μmol (photon) m^–2^), bottom incident light accumulation (I_BI_, μmol (photon) m^–2^), and light interception accumulation (I_I_, μmol (photon) m^–2^).

**Table 2:** The chlorophyll fluorescence characteristics of peanut.

Year	Treatment		Fv/Fm	ΦPSⅡ	qP	qN	ETR
2019	P		0.762 ± 0.016b	0.369 ± 0.035b	0.332 ± 0.004b	0.840 ± 0.014a	180.188 ± 2.946a
	The middle row	I	0.782 ± 0.014ab	0.448 ± 0.010a	0.465 ± 0.004a	0.757 ± 0.008b	147.393 ± 6.231c
		RI	0.777 ± 0.014ab	0.462 ± 0.022a	0.464 ± 0.006a	0.765 ± 0.032b	145.534 ± 4.614c
		RI2	0.767 ± 0.014b	0.466 ± 0.023a	0.468 ± 0.009a	0.771 ± 0.031b	143.684 ± 6.427c
		RI3	0.784 ± 0.005ab	0.466 ± 0.013a	0.467 ± 0.004a	0.789 ± 0.019b	146.515 ± 4.607c
	The border row	I	0.783 ± 0.024ab	0.473 ± 0.032a	0.472 ± 0.007a	0.784 ± 0.03b	158.086 ± 2.886b
		RI	0.806 ± 0.007a	0.474 ± 0.025a	0.471 ± 0.008a	0.791 ± 0.037b	160.764 ± 5.467b
		RI2	0.810 ± 0.009a	0.479 ± 0.002a	0.468 ± 0.009a	0.742 ± 0.027b	159.129 ± 10.925b
		RI3	0.800 ± 0.016ab	0.472 ± 0.016a	0.469 ± 0.002a	0.761 ± 0.026b	159.505 ± 4.812b
2020	P		0.766 ± 0.021b	0.388 ± 0.020b	0.418 ± 0.025c	0.782 ± 0.007a	169.632 ± 7.362a
	The middle row	I	0.777 ± 0.004ab	0.448 ± 0.010a	0.463 ± 0.01b	0.715 ± 0.015b	124.299 ± 5.698d
		RI	0.769 ± 0.008b	0.462 ± 0.022a	0.491 ± 0.004a	0.66 ± 0.01c	139.071 ± 4.99bc
		RI2	0.763 ± 0.011b	0.466 ± 0.023a	0.492 ± 0.01a	0.661 ± 0.01c	137.034 ± 2.627bc
		RI3	0.777 ± 0.017ab	0.466 ± 0.013a	0.497 ± 0.002a	0.654 ± 0.015c	134.623 ± 3.071cd
	The border row	I	0.779 ± 0.014ab	0.473 ± 0.032a	0.494 ± 0.008a	0.68 ± 0.007c	144.633 ± 3.345bc
		RI	0.800 ± 0.013ab	0.474 ± 0.025a	0.502 ± 0.007a	0.649 ± 0.014c	148.724 ± 3.54b
		RI2	0.806 ± 0.012a	0.488 ± 0.018a	0.498 ± 0.014a	0.668 ± 0.027c	149.681 ± 5.522b
		RI3	0.806 ± 0.014a	0.472 ± 0.016a	0.503 ± 0.002a	0.659 ± 0.017c	149.016 ± 4.296b

P, sole peanut; I, maize–peanut intercropping; RI, maize–peanut–intercropping, RI2, 20% N reduction for maize of RI; ICN3, 40% N reductions for maize of RI.

The parameters are: Fv/Fm (optimal/maximal photochemical efficiency of PSII in the dark), ΦPS II (actual photochemical efficiency of PS II in the light), qP (Photochemical quenching), qN (Non-photochemical quenching) and ETR (electron transport rate). Different lowercase letters represent signifificant (P<0.05).

Rotating the peanut and maize planting strips had no effect on peanut light interception, but significantly impacted light response curve (**Table 1**). In 2020, after rotating the planting strip, intercropped border row peanut had a significant 10.55% increase in Pn_max_ and a 7.76% decrease in I_sat_. Similar phenomenon was not observed in 2019. And in 2020, a significant increase in qP and ETR and a significant decrease in qN in the intercropping border row were found after rotating the planting strip. In this experiment, N reduction for maize in the maize–peanut intercropping systems had no significant effect on the incident light, photosynthetic parameters and fluorescence parameters of intercropped peanut.

### 3.3 Photosynthetic character of maize

In the maize–peanut intercropping systems, maize is the dominant crop due to its higher plant height. The incident light in the top and middle of intercropped maize was always higher than that of sole maize before 16:00 and 14:00, while the bottom incident light was always higher than that of sole maize (**Figures S3**, **S4**). The incident light in the top and spike leaf of intercropped border row maize was higher than those of the middle row maize before 13:30. During the testing period, the intercropped border and middle row maize compared to the sole maize showed an average increase in top incident light accumulation by 6.05% and 5.54%, spike leaf incident light accumulation by 64.04% and 52.03%, bottom incident light accumulation by 53.70% and 18.80%, light interception accumulation by 11.53% and 4.84% (**Table 3**). Pn, Ci, Pn_max_, ΦPSII, and ETR increased and Rd decreased in intercropped maize compared to sole maize (**Figure 3** and **Table 3**).

**Table 3:** The chlorophyll fluorescence characteristics of maize.

Year	Parameters	M	I		RI		RI2		RI3	
BR	MR	BR	MR	BR	MR	BR	MR			
2019	Rd	5.24	6.00	5.74	5.86	5.53	5.73	5.46	5.62	5.59
	I_comp_	119.16	136.96	132.22	141.03	133.96	134.97	135.11	137.38	121.94
	I_sat_	1773.63	1768.51	1778.92	1820.63	1762.28	1776.27	1763.38	1789.06	1684.72
	Pn_max_	41.95	47.25	45.24	46.62	45.42	46.03	44.81	45.71	43.46
	R^2^	99.63%	99.51%	99.63%	99.70%	99.73%	99.86%	99.80%	99.83%	99.44%
	I_TI_	21293.21	23697.65	22575.53	23967.74	22565.57	23858.00	21997.08	23529.34	22529.50
	I_PI_	2571.05	4965.91	4378.37	4942.22	4345.35	5051.74	4419.44	5084.67	4466.50
	I_BI_	1940.76	2564.30	2467.49	2464.24	2346.37	2517.96	2391.01	2558.03	2429.45
	I_I_	18698.04	20472.36	19459.33	20803.46	19544.64	20674.77	18974.54	20309.53	19429.68
2020	Rd	5.23	6.00	5.87	6.24	5.80	6.10	5.72	6.23	5.45
	I_comp_	135.98	148.45	150.56	153.60	146.96	152.95	151.30	152.53	123.00
	I_sat_	1781.62	1793.32	1794.83	1783.12	1786.92	1795.22	1772.45	1784.93	1747.73
	Pn_max_	43.07	46.54	45.16	46.02	44.78	45.87	43.52	44.77	41.48
	R^2^	99.59%	99.38%	99.60%	99.36%	99.60%	99.59%	99.75%	99.41%	99.64%
	I_TI_	21396.48	23731.01	22697.79	23679.83	22365.27	24019.29	22704.76	23419.74	22230.69
	I_PI_	3091.13	5417.59	4877.30	5341.33	4802.37	5514.74	4924.01	5491.01	4884.41
	I_BI_	2608.65	2962.89	2881.67	2993.10	2894.30	3084.18	2989.68	2890.24	2790.90
	I_I_	18291.11	20252.74	19318.72	20174.34	18965.04	20408.13	19228.38	20028.28	18925.15

M, sole maize; I, maize–peanut intercropping; RI, maize–peanut–intercropping, RI2, 20% N reduction for maize of RI; ICN3, 40% N reductions for maize of RI.

The parameters are: Dark respiration rate (R_d_, μmol (CO_2_) m^–2^ s^–1^), light saturation point (I_sat_, μmol (photon) m^–2^ s^–1^), light compensation point (I_comp_, μmol (photon) m^–2^ s^–1^), asymptotic estimate of the maximum gross photosynthetic rate (P_gmax_, μmol(CO_2_) m^–2^ s^–1^). The light includes top incident light (I_TI_, μmol (photon) m^–2^ s^–1^) and bottom incident light (I_DI_, μmol (photon) m^–2^ s^–1^). The PAR includes top incident light accumulation (I_TI_, μmol (photon) m^–2^), spike leaf incident light accumulation (I_PI_, μmol (photon) m^–2^), bottom incident light accumulation (I_BI_, μmol (photon) m^–2^), and light interception accumulation (I_I_, μmol (photon) m^–2^).

Rotating the planting strip and a 20% N reduction had no significant effect on the photosynthetic character of intercropped maize (**Figure 3** and **Table 3**). A 40% N reduction led to a significant reduction in Pn by 7.86% and 13.03%, 11.92% and 12.27% in the intercropped middle row and border row in 2019 and 2020 respectively. For intercropped middle row maize, the Gs, Pn_max_, I_comp_, qP, ΦPSII, and ETR significantly decreased after a 40% N reduction; but for intercropped border row maize, only the decrease of qP was found to significantly decreased.

### 3.4 Rubisco-related enzyme activity

**Figure 4:**
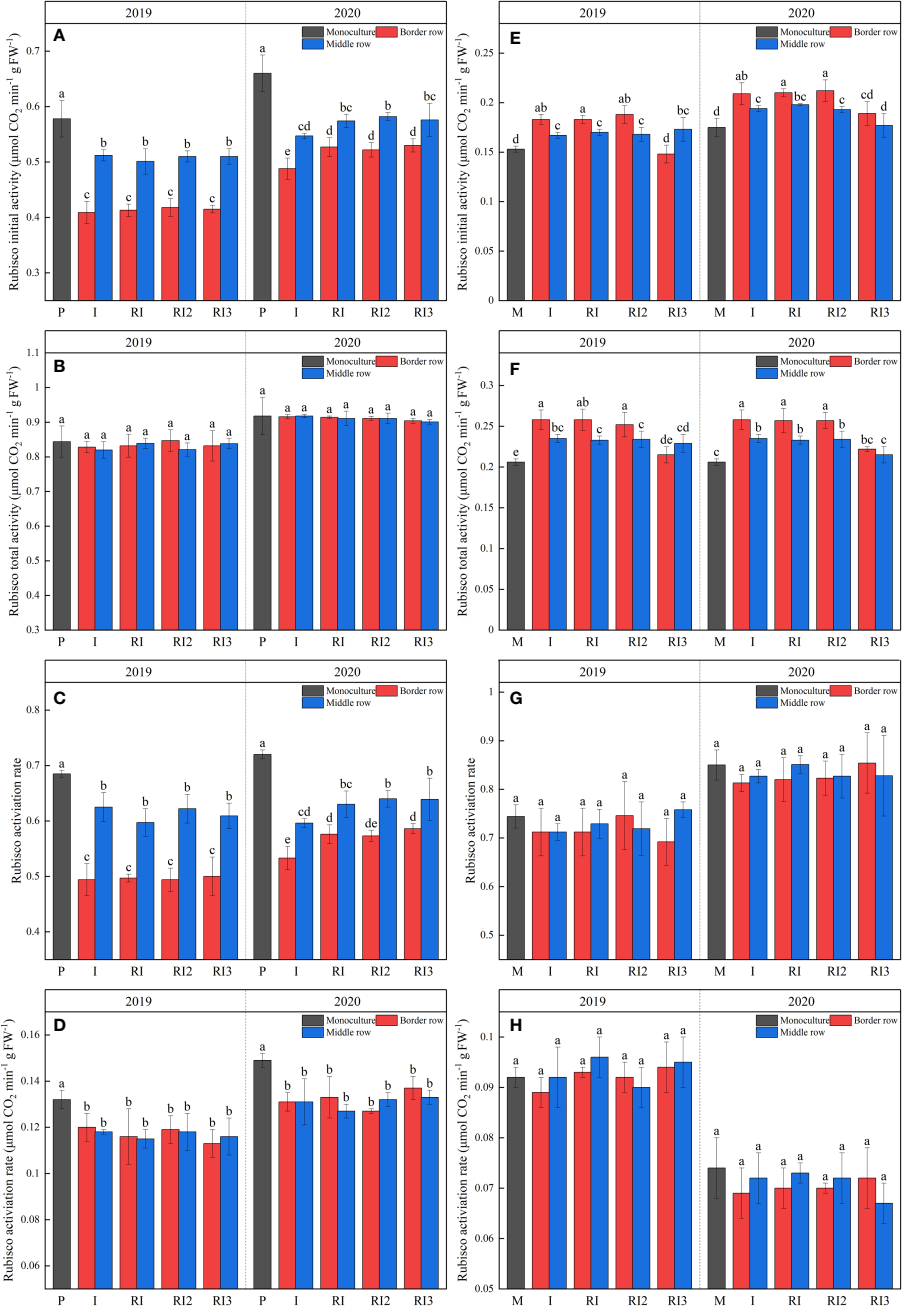
Rubisco initial activity **(A, E)**, Rubisco total activity **(B, F)**, Rubisco activation rate **(C, G)**, and Rubisco activase **(D, H)** of peanuts and maize. P, sole peanut; M, sole maize; I, maize–peanut intercropping; RI, maize–peanut rotation–intercropping, RI2, 20% N reduction for maize of RI; ICN3, 40% N reductions for maize of RI. Different lowercase letters represent significant (P<0.05).

Rubisco is the key enzyme that catalyzes the initial steps of photosynthetic CO_2_ fixation. After intercropping, Rubisco initial activity, Rubisco activation rate, and RCA were significantly reduced in peanuts, with the reduction being much greater in the intercropped border row than in the intercropped middle row (**Figure 4**). Rotation strips did not affect Rubisco total activity and Rubisco activation rate in intercropped peanut, but significantly increased the Rubisco initial activity by 7.85% and Rubisco activation rate by 7.97% respectively in intercropped border row peanut in 2020. N reduction had no significant effect on Rubisco-related enzyme activity for peanut.

After intercropping, Rubisco initial activity and Rubisco total activity in the intercropped maize increased significantly due to light dominance (**Figure 4**). Rotating the planting strip does not affect maize Rubisco-related enzyme activity. A 40% N reduction reduced Rubisco initial activity and Rubisco total activity in the intercropped maize. It is noteworthy that Rubisco initial activity and Rubisco total activity were significantly higher in the I, RI, and RI2 border row than in the middle row, while this phenomenon was not observed in RI3.

### 3.5 Yield and yield-related traits

**Figure 5:**
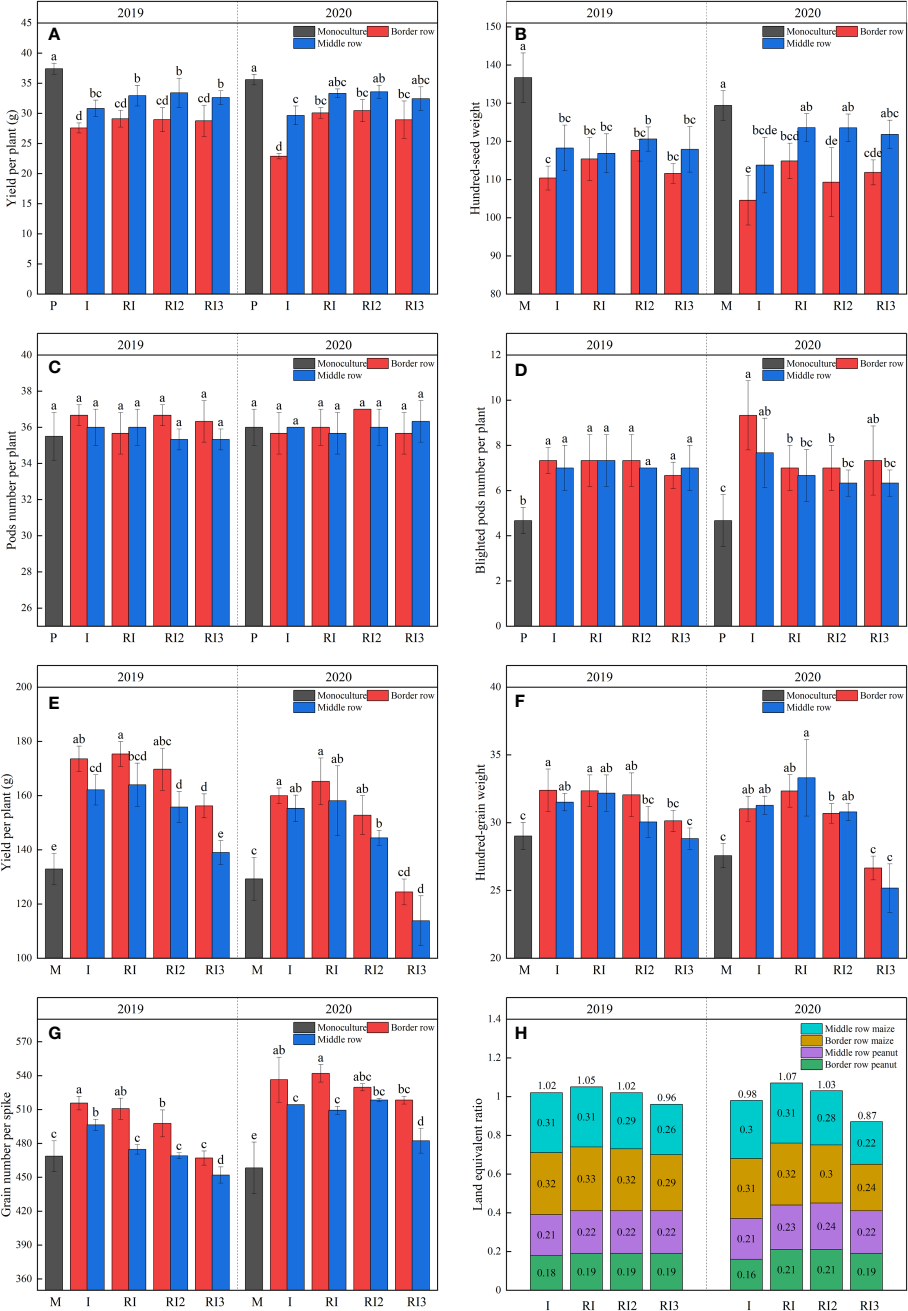
Yield, yield–related traits and land equivalent ratio. **(A)**, yield per plant of peanut; **(B)**, hundred-grain weight of peanut; **(C)**, full pods number per plant of peanut; **(D)**, blighted pods number per plant of peanut; **(E)**, yield per plant of maize; **(F)**, hundred-grain weight of maize; **(G)**, grain number per spike of maize; **(H)** land equivalent ratio. P, sole peanut; M, sole maize; I, maize–peanut intercropping; RI, maize–peanut rotation–intercropping, RI2, 20% N reduction for maize of RI; ICN3, 40% N reductions for maize of RI. Different lowercase letters represent significant (P<0.05).

Intercropping peanut is a disadvantageous crop. After intercropping, peanuts pods number per plant remains unchanged, while the hundred-grain weight (HGW) decrease significantly and blighted pods number per plant (N_BP_) increase significantly, resulting in 26.26% and 17.58%, 35.71% and 16.67% reduction in yield per plant in 2019 and 2020 for intercropped border and middle rows, respectively (**Figure 5**). After rotating the planting strips, there was no significant difference in peanut yield per plant in 2019, but the yield per plant of intercropped border row peanut increased by 27.70% in 2020. Significant decreases in yield per plant and HGW were observed in sole peanut in 2020 compared to 2019. Importantly, the decreases in yield per plant and HGW of intercropped peanut in maize–peanut intercropping but not in maize–peanut rotation–intercropping. N reduction did not affect peanut yield and yield-related traits.

After intercropping, the yield per plant, HGW, and grain number per spike increased significantly in the intercropped maize (**Figure 5**). Rotating the planting strips and a 20% N reduction does not affect maize yield and yield-related traits. But after reducing N by 40%, the yield per plant, grain number per spike, and HGW of maize decreased significantly. Interannual variation in maize yield and yield-related properties for all treatments was not significant in 2019 and 2020.

The LER for all the intercropping treatments ranged from 0.88 to 1.07. LER for maize–peanut intercropping in 2019 and 2020 were 1.02 and 0.98. In 2019, rotating the planting strip increased the LER by 2.94%, but in 2020 it increased the LER by 9.18% by increasing the peanut yield in intercropped border row. A 40% N reduction for maize can reduce the LER of intercropping below 1 by decreasing the maize yield. Significant reductions in LER were observed in maize–peanut intercropping compared to 2019, while this was not found in maize–peanut rotation–intercropping.

## 4 Discussion

### 4.1 The photosynthetic responses of peanut adaptation for intercropping

The degree of interspecific competition between different crops affects the morphology and yield formation of crops within the intercropping systems (Lv et al., 2014). In this study, there are two primary factors that influence the photosynthesis of intercropped peanut, one is an increase in soil N in the intercropped peanut planting strip caused by top dressing in the maize planting strips (**Figure 2**), and the second is light disadvantage (**Table 1**) by tall intercropped maize. Videlicet, the increase in soil nitrogen for intercropped peanuts cannot compensate for the loss of light. A significant reduction was measured for incident light interception and Pn in intercropped peanut (**Figure 3**). In addition, the Ci of intercropped peanut were also found to be significantly lower, which also indicates that lower CO_2_ concentrations were also important in reducing the effects of photosynthesis in intercropped peanuts. This may be attributable to tall intercropped maize affecting air movement (especially CO_2_), especially for C_3_ crops such as peanut with a low affinity of Rubisco for CO_2_ (von Caemmerer and Quick, 2000).

Plants are highly light-adapted, and changes in the light conditions received by the intercropping population under intercropping systems have led to significant changes in the photosynthetic character of the crop. In general, plant leaves grown in shade condition was thinner and large, had a thinner fence tissue thickness and chloroplast (Terashima et al., 2006; Gong et al., 2015), the more light-harvesting complexes per unit, a lower CO_2_ assimilation rate saturated (Huang et al., 2011; Zhu et al., 2012). The light response curve can reflect the photosynthetic character of plants. According to Yao et al. (2017), the intercropped soybean (*Glycine max* L.) showed a 16.91%, 40.00%, and 9.62% reduction in Pn_max_, I_comp_, and I_sat_, and a 30.41% increase in Rd compared to the sole soybean. In this study, similar results for I_comp_, I_sat_, and Rd were obtained for intercropped peanut, but not the Pn_max_ (**Table 1**). This suggests that the maximum photosynthetic potential of peanut is not inhibited, which would be different from soybean. Previous research has shown that Peanuts are extremely sensitive to soil Fe content. A shortage of Fe drastically disrupted photosynthesis, and caused a marked reduction in small and large subunits of Rubisco content, chlorophyll a/b-binding proteins content, and RNA synthesis in leaf (Abadía, 1992; Winder and Nishio, 1995). Therefore, we suspected that the absence of a decrease in Pn_max_ in intercropped peanut is attributed to the improvement in iron uptake caused by the low Fe requirement of intercropped maize than peanut (Zuo et al., 2000).

Rubisco is the key enzyme to determine the direction and efficiency of photosynthetic carbon metabolism in plants (Andersson and Backlund, 2008), and the initial and total Rubisco activity are widely used to estimate both *in vivo* activation state and full carboxylation potential (Paulilo et al., 1994; Parry et al., 1997). In past reports, researchers have confirmed that the Rubisco activity is significantly affected by changes in light and decreases with the reduction in light availability (Paulilo et al., 1994; Andersson and Backlund, 2008). In this study, intercropped peanuts showed a significant reduction in carboxylation rates under light stress, but still maintained a strong full carboxylation potential (**Figures 3**, **4**), which is also consistent with the low Pn and constant Pn_max_. Intercropped peanuts showed substantial potential to increase yield. The higher carbon sequestration potential of photosynthetic enzymes in intercropped peanut is also reported in the protein analysis of Xiong et al. (2013). The low Rubisco activation state of intercropped peanut was due to a decrease in RCA activity caused by light stress (Butz and Sharkey, 1989; Andersson and Backlund, 2008), while RCA is necessary for catalytic Rubisco (Parry et al., 1997).

During photosynthesis, the function of the photosystem II (PSII) reaction center is crucial for limiting light energy use and the proper functioning of photosynthesis, and its activity is particularly sensitive to light stress (Schreiber et al., 1995). Compared with sole peanut, intercropped peanuts had higher electron transport efficiency and effective quantum efficiency of PSII to adapt to less light. However, these light-adapted responses still could not compensate for the light inhibition, and the electron transport rate was significantly reduced. This is also an important reason for the reduction of the photosynthetic rate of intercropping peanut.

### 4.2 The photosynthetic responses of maize adaptation for cropping system

As a cereal crop with a high stem, high-water-consuming, deep roots system C_4_ crop, intercropped maize has always been in a dominant ecological position in the maize–peanut intercropping (Lv et al., 2014; Li et al., 2019). At the same time, in this study, intercropped maize was subjected to a long period of N stress after top dressing (**Figure 2**) (due to a soil N transfer from MPS to PPS). Obviously, the growth advantages received by intercropped maize can compensate for the soil N disadvantage, and the Pn and yield per plant of intercropped maize were significantly greater than those of sole maize (**Figure 5**). In the study of Lv et al. (2014), competition for nutrients was of more importance than the competition for light through root separation experiment in maize–soybean intercropping, which doesn’t correspond to our study. A possible explanation for this result is that maize growth was not subjected to sufficient N stress to affect growth in this study due to (i) the sufficient water and light advantages received by intercropped maize than sole maize can increase the uptake and use of soil N (Sims et al., 1998a; Good et al., 2004); (ii) a large amount of N lost through leaching and nitrous oxide in dry farming areas soil (Shaver et al., 2002; Gao et al., 2009); (iii) the underlying nutrient sharing by peanut with maize (Searle et al., 1981). Several scientists have claimed that intercellular CO_2_ concentration and stomatal conductance are correlated, but not in this study, which may be a response strategy for maize to avoid excessive transpiration in the face of water deficiency in arid agricultural areas. This also suggests that stomata are not a factor for the increased photosynthetic rate (Yang et al., 2017).

Increasing Pn_max_ and I_comp_ were common responses of many plants, including most crop species, to higher light, having the effect of improving the efficiency of carbon assimilation (Givnish et al., 2004; Pfanz et al., 2007). Similar results were found in intercropped maize. The higher photosynthetic rates and photosynthetic potential of intercropped maize compared to sole maize are mainly due to the high carboxylation rates, light energy conversation efficiency primary, and electron transport rate (**Table 4**). The carboxylation capacity of intercropped maize increased by increasing initial Rubisco activity rather than total Rubisco activity, and the I_sat_ of intercropped maize was not found to increase, which may be due to the soil N content limitation.

**Table 4:** The chlorophyll fluorescence characteristics of maize.

Year	Treatment		Fv/Fm	ΦPSⅡ	qP	qN	ETR
2019	M		0.696 ± 0.031a	0.219 ± 0.003e	0.337 ± 0.019a	0.719 ± 0.046a	116.35 ± 9.325d
	The border row	I	0.697 ± 0.030a	0.342 ± 0.01abc	0.338 ± 0.009a	0.721 ± 0.032a	157.126 ± 13.966a
		RI	0.709 ± 0.039a	0.354 ± 0.007ab	0.339 ± 0.003a	0.747 ± 0.046a	154.918 ± 8.066ab
		RI2	0.685 ± 0.015a	0.332 ± 0.028abc	0.329 ± 0.010a	0.752 ± 0.046a	154.562 ± 6.768ab
		RI3	0.681 ± 0.019a	0.361 ± 0.015a	0.305 ± 0.008b	0.743 ± 0.032a	155.717 ± 6.513ab
	The middle row	I	0.658 ± 0.014a	0.320 ± 0.009c	0.341 ± 0.010a	0.717 ± 0.031a	144.114 ± 9.287ab
		RI	0.651 ± 0.008a	0.326 ± 0.021bc	0.318 ± 0.014a	0.763 ± 0.018a	145.003 ± 9.332ab
		RI2	0.680 ± 0.015a	0.337 ± 0.024abc	0.341 ± 0.009a	0.754 ± 0.024a	139.438 ± 6.777bc
		RI3	0.719 ± 0.014a	0.292 ± 0.010d	0.279 ± 0.018b	0.744 ± 0.033a	125.904 ± 5.66cd
2020	M		0.740 ± 0.021a	0.244 ± 0.008d	0.361 ± 0.019ab	0.838 ± 0.009a	138.007 ± 7.54d
	The border row	I	0.752 ± 0.009a	0.344 ± 0.013a	0.353 ± 0.012ab	0.797 ± 0.008a	164.787 ± 6.527abc
		RI	0.769 ± 0.014a	0.328 ± 0.005ab	0.367 ± 0.021a	0.818 ± 0.017a	172.468 ± 7.677ab
		RI2	0.751 ± 0.014a	0.322 ± 0.009ab	0.358 ± 0.019ab	0.817 ± 0.018a	171.407 ± 1.793ab
		RI3	0.753 ± 0.032a	0.325 ± 0.015ab	0.328 ± 0.007bc	0.815 ± 0.033a	174.882 ± 6.019a
	The middle row	I	0.751 ± 0.033a	0.317 ± 0.013ab	0.338 ± 0.004ab	0.820 ± 0.026a	155.194 ± 4.455c
		RI	0.745 ± 0.024a	0.327 ± 0.02ab	0.346 ± 0.019ab	0.805 ± 0.026a	160.225 ± 7.473bc
		RI2	0.756 ± 0.016a	0.302 ± 0.021bc	0.351 ± 0.037ab	0.835 ± 0.016a	154.936 ± 6.666c
		RI3	0.743 ± 0.016a	0.283 ± 0.015c	0.299 ± 0.009c	0.834 ± 0.035a	133.741 ± 11.407d

M, sole maize; I, maize–peanut intercropping; RI, maize–peanut rotation–intercropping, RI2, 20% N reduction for maize of RI; ICN3, 40% N reductions for maize of RI.

The parameters are: Fv/Fm (optimal/maximal photochemical efficiency of PSII in the dark), ΦPS II (actual photochemical efficiency of PS II in the light), qP (Photochemical quenching), qN (Non-photochemical quenching) and ETR (electron transport rate). Different lowercase letters represent signifificant (P<0.05).

### 4.3 The photosynthetic responses of peanut and maize adaptation after rotating planting strips

After harvest, leguminous crops leave a lot of root nodules and residues as organic N for the following crop (Fischer et al., 2002). Crops grown after legumes tend to have a high Pn and yield. Contrary to our hypothesis that there were no significant differences between intercropped maize after rotating the planting strips compared to maize in the non-rotating planting strips (**Tables 3**, **4**). The most probable explanation is that organic N are consumed during the winter fallow period (McGuire et al., 1998). As this study was only carried out for three years, long-term maize–peanut rotation–intercropping is expected to improve soil quality and intercropped maize yields.

Peanut is highly susceptible to continuous cropping obstacles due to soil nutrient imbalance, allelopathy, microbial community imbalance and increased soil pathogenic microorganisms (Li et al., 2020). The main factor affecting the photosynthesis of intercropped peanut is the soil after the rotation of the planting strips because the growth of intercropped maize is not affected. After rotating the planting strips, in the second planting year (2019), the photosynthetic rate and yield of intercropped peanut did not change significantly (**Table 1** and **Figures 3**, **5**). But in the third planting year (2020), after rotating the planting strips, the Pn_max_, Pn and yield per plant of intercropping border row peanut increased significantly. On the one hand, this indicated that intercropped fringe border peanut would suffer from a continuous cropping obstacle enough to affect Pn and yield in the third planting year, and on the other hand, it confirmed that rotating the planting strip in maize–peanut intercropping can enhance the resistance of peanuts to continuous cropping obstacles. The increase in Pn and yield are the consequence of the combined increase in carboxylation rates, electron transport rate, and electron transport efficiency (**Tables 1**, **2**). In addition, continuous cropping obstacle primarily occurs in intercropped border row rather than in intercropping middle row, it also shows that better light radiation can alleviate the continuous crop barrier.

### 4.4 The photosynthetic responses of peanut and maize adaptation after N fertilizer reduction

N is an essential nutrient and in addition it is a basic input in dry farming. Generally, N fertilization helps reduce the intraspecific competition between intercrops (Zhang et al., 2021). To achieve the maximum economic benefit, N fertilizer rates need to be carefully adjusted (Parihar et al., 2017; Yong et al., 2018) In this study, N reduction did not affect the light interception in intercropped maize and peanut (**Table 3**), and the factor affecting crop growth was the change in soil N content. Similarly, although the N reduction was only in the maize planting strip, the peanut planting strip was necessarily subject to N stress due to N transport in the soil. However, in this study, no effect of this N stress on photosynthesis and yield of peanut was observed. The most probable explanation was the low soil N requirement of intercropped peanuts.

Maize is more sensitive to soil N stress. A 20% N reduction had no effect on the photosynthetic character, Rubisco-related enzyme activity, and yield of intercropped maize (**Tables 3**, **4** and **Figures 3**–**5**), indicating that maize was not under sufficient N stress to affect normal photosynthesis and production. This is attributed to: (i) a 20% N reduction had a small and short-term effect on soil N content and as not sufficient to affect photosynthesis in maize (**Figure 2**); (ii) the increased N uptake and utilization capacity of intercropped maize can compensate for the reduced soil N content (Sims et al., 1998a; Gao et al., 2009); (iii) intercropped maize can use the nitrogen fixed by peanuts. After a 40% N reduction, the N compensating and light utilization ability based on intercropping advantage was limited (**Table 3**). The reduction in photosynthesis in intercropped maize is a combination of reduced Gs and carboxylation capacity, light conversion capacity and electron transfer rate. It is normal that the synthesis of Rubisco requires the involvement of N (Andersson and Backlund, 2008). The increase of initial and total Rubisco activity in intercropped border row maize than in intercropped middle row was not found after 40% N reduction, but in the 20% N reduction and non-reduction treatments, which is important evidence that the rate of carboxylation in intercropped maize is more strongly N limited.

### 4.5 Yield under different planting patterns and N application rates

The main reason for farmers to implement intercropping is that it can improve land productivity with a small investment (Zhang et al., 2021). There is conflicting evidence regarding the advantages of the land-use advantage of maize–peanut intercropping (Feng et al., 2021). These apparent discrepancies may arise from the fact that the different studies were performed under different environmental conditions and border–row proportions (Wang et al., 2020; Feng et al., 2021). In our study, the LER for maize–peanut intercropping in 2019 and 2020 was 1.02 and 0.98, respectively, indicating that there are no benefits from land use efficiency in dry farming areas (**Figure 5**). The increase in maize yield was attributable to an increase in HGW and grain number per spike, while the decrease in peanut yield was mainly attributable to a decrease in HGW and an increase in the N_BP_.

After rotating the planting strip, intercropped border row yield increased significantly in maize–peanut rotation–intercropping than in maize–peanut intercropping due to an increase in hundred-grain weight (HGW) and blighted pods number per plant (N_BP_). This supports our conclusion that the rotation of the planting strip can alleviate the continuous crop barrier of peanuts in the maize–peanut intercropping. Although the increase in productivity of intercropped middle row peanut in maize–peanut rotation–intercropping did not reach significant levels (*p* ≤ 0.05), it was evident that yield per plant, HGW and N_BP_ in the intercropped border rows peanut had also reached the level of sole peanut. This is also evidence of the alleviation of the continuous crop barrier in the intercropped middle row peanut. More importantly, in both 2019 and 2020, the LER for maize–peanut rotation–intercropping was greater than 1, showing a significant land-use advantage. A 20% N reduction had no effect on LER of maize–peanut rotation–intercropping in dry farming areas. A 40% N reduction in the maize planting strip reduced the LER to less than 1, and decreased by 8.33% in 2020 compared with 2019, owing to lower HGW and grain number per spike of intercropped maize. The lower LER in 2020 can be explained by excessively reduced N application leading to a long-term lower N input to farmland than N output, resulting in lower soil farm fertility. Hereby, to improve photosynthetic production capacity and economic benefits in dry farming areas, first of all, optimize cropping patterns and choose maize–peanut rotation–intercropping to improve the photosynthetic production capacity of maize and alleviate the continuous crop barriers of peanut; secondly, reduce nitrogen without affecting maize growth.

## 5 Conclusions

In maize–peanut intercropping, the intercropped peanut adapts to light inhibition by decreasing light saturation point (I_sat_), reducing light compensation point (I_comp_), and increasing electron transport efficiency, but exhibits low intercellular CO_2_ concentration (Ci), carboxylation rates, and electron transport rate, resulting in lower hundred-grain weight (HGW) and higher blighted pods per plant. For intercropped maize, Ci, electron transfer rate, electron transfer rate, Pn, maximum photosynthetic rate (Pn_max_) and I_comp_ increased simultaneously, resulting in higher HGW and grain number per spike. But there was no land-use advantage to maize-peanut intercropping in dry farming areas. Rotating the planting strips in maize–peanut intercropping (maize–peanut rotation–intercropping) had no effect on intercropped maize, but mitigated succession barriers in intercropped border peanut through increased carboxylation rates, electron transport rate and transport efficiency, which in turn leads to an increase in the yield of intercropped peanuts per plant and an improvement in farmland land use efficiency. In maize–peanut rotation–intercropping, a moderate N reduction has no significant effect on photosynthetic capacity and yield of peanuts and maize, but excessive N reduction decreased the carboxylation rates, photoconversion ability, electron transport rate and Pn_max_ in intercropped maize, leading to a significant reduction in Pn, HGW and grain number per spike, as well as land equivalent ratio. Therefore, maize–peanut rotation–intercropping with N reduction for maize can be recommended as an excellent cropping system to improve agricultural productivity in dry farming areas. The method and results of this study can be generalized to provide guidance to the optimization of cropping systems in dry farming areas.

## Data availability statement

The original contributions presented in the study are included in the article/**Supplementary Material**, further inquiries can be directed to hanfeiyanzhou@163.com.

## Author contributions

XC and XR: Conceptualization. SG: Methodology. SW: software. XC and XR: Validation. RG and SG: Formal analysis. FH and ZJ: Investigation. TC and PZ: Resources. FH: Data curation. FH: Writing-Original draft preparation. XC, XR, SH, TJ and PS: Writing-Reviewing and Editing. XC and XR. Project administration. BP and PS: Funding acquisition. All authors have read and agreed to the published version of the manuscript.

## Funding

We thank the Program of Shaanxi Province Key R&D Program (No. 2021NY-073 and No. 2022NY-196), National Natural Science Foundation of China (No. 31871580, 31871562), Key R&D program of Ningxia Hui Autonomous Region (No. 2019BBF03011) for generous financial support. We also thanks Researchers Supporting Project Number (RSP2022R410), King Saud University, Riyadh, Saudi Arabia for funding. The Article Processing Charge is financed by Wroclaw University of Environmental and Life Science. The Tax Idenfication Number (NIP) is PL8960005354.

## Acknowledgments

The authors would like to extend their sincere appreciation to the Researchers Supporting Project Number (RSP2022R410), King Saud University, Riyadh, Saudi Arabia.

## Conflict of interest

The authors declare that the research was conducted in the absence of any commercial or financial relationships that could be construed as a potential conflict of interest.

## Publisher’s note

All claims expressed in this article are solely those of the authors and do not necessarily represent those of their affiliated organizations, or those of the publisher, the editors and the reviewers. Any product that may be evaluated in this article, or claim that may be made by its manufacturer, is not guaranteed or endorsed by the publisher.
