# Supplementary material for: Photosynthetic and yield responses of rotating planting strips and reducing nitrogen fertilizer application in maize–peanut intercropping in dry farming areas
Source: Front Plant Sci. 2022 Nov 16;13:1014631. doi: 10.3389/fpls.2022.1014631 (PMC9708908; doi:10.3389/fpls.2022.1014631)
Supplement: Supplementary file 1 [file DataSheet_1.docx]

Supplementary Material

**Supplementary Figure 1.** Incident PAR at top and bottom of peanuts in 2019 and 2020. P, sole peanut; M, sole maize; I, maize–peanut intercropping; RI, maize–peanut rotation–intercropping, RI2, 20% N reduction for maize in RI; ICN3, 40% N reductions for maize in RI.

**Supplementary Figure 2.** Light responses of net photosynthetic rate (Pn) of peanut. a and d: sole peanut (P) and maize–peanut intercropping (I) in 2019 and 2020; b and e: I and rotation–intercropping (RI) in 2019 and 2020; c and f: RI and 20% (RI2) and 40% (RI3) N reductions for maize in RI. LRC means the light response curve.

**Supplementary Figure 3.** Incident PAR at top and bottom of maize in 2019 and 2020. M, sole maize; I, maize–peanut intercropping; RI, maize–peanut rotation–intercropping, RI2, 20% N reduction for maize in RI; ICN3, 40% N reductions for maize in RI.

 **Supplementary Figure 4.** Light responses of net photosynthetic rate (Pn) of maize. a and d: sole maize (M) and maize–peanut intercropping (I) in 2019 and 2020; b and e: I and rotation–intercropping (RI) in 2019 and 2020; c and f: RI and 20% (RI2) and 40% (RI3) N reductions for maize in RI. LRC means the light response curve.
